# Alternative Sensor System and MLP Neural Network for Vehicle Pedal Activity Estimation

**DOI:** 10.3390/s100403798

**Published:** 2010-04-14

**Authors:** Ahmed M. Wefky, Felipe Espinosa, José A. Jiménez, Enrique Santiso, José M. Rodríguez, Alfredo J. Fernández

**Affiliations:** Electronics Department, Polytechnics, University of Alcalá, Campus Universitario s/n, 28871 Alcalá de Henares, Madrid, Spain; E-Mails: awefky@depeca.uah.es (A.M.W.); espinosa@depeca.uah.es (F.E.); santiso@depeca.uah.es (E.S.); jmra@depeca.uah.es (J.M.R.); alfredo.fernandezr@uah.es (A.J.F)

**Keywords:** brake estimator, clutch estimator, throttle estimator, pedals activity estimation, multilayer perceptron, ANN estimator, on-board sensorial system

## Abstract

It is accepted that the activity of the vehicle pedals (*i.e.*, throttle, brake, clutch) reflects the driver’s behavior, which is at least partially related to the fuel consumption and vehicle pollutant emissions. This paper presents a solution to estimate the driver activity regardless of the type, model, and year of fabrication of the vehicle. The solution is based on an alternative sensor system (regime engine, vehicle speed, frontal inclination and linear acceleration) that reflects the activity of the pedals in an indirect way, to estimate that activity by means of a multilayer perceptron neural network with a single hidden layer.

## 1. Introduction

Driving is one of the essentials in our daily life. Nowadays there are strong research efforts invested in the modeling of the driver behavior using driving signals by means of hidden Markov models (HMMs) [1,2] and dynamic belief networks (DBN) [3] as well as predicting the future status of the vehicle, drowsy or drunk driving detection with eye monitoring [4,5], and the cognitive modeling of drivers [6]. Another research trend is to recognize the driver activity (*i.e.*, operating the navigation system, adjusting the mirrors, talking to other passengers... *etc.*) using force sensor arrays (FSAs) that measure the driver sitting postures [7]. In this paper, the authors concentrate on presenting a neural predictor that is able to estimate the pedals action as a means to conclude the driver activity related to the driving dynamics. As can be noticed from the previous works in the same field, it is complex to separate the theoretical aspects concerning the driver behavior from the experimental ones [1–7].

Observable and measurable driving signals can be divided into three categories [8]: (1) driving activity signals, e.g., throttle, clutch and brake pedal activities or pressures and steering wheel angles; (2) vehicle state signals, e.g., vehicle speed and acceleration, regime engine; and (3) vehicle position signals, e.g., following distance, relative lane position, and relative angles (pitch and roll).

The instantaneous and simultaneous measurement of the three car pedals is a difficult task in the great majority of vehicles. In fact, there are 3 methods to directly measure the status of the car pedals: using position sensors [9], using pressure sensors [10–14] and through the electronics system provided by the vehicle manufacturer (e.g., SMART and RAVEL-I [2,15]).

**Figure 1.**
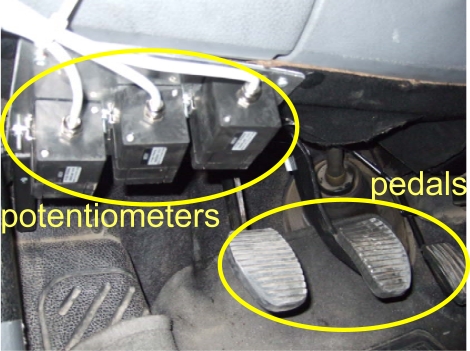
Example of potentiometers used as position sensors for vehicle pedal activity.

The authors of this paper have the design experience of a universal and non-disturbing solution (Miveco Resesarch Project [9]) to register the displacement of the pedals by means of potentiometers as shown in Figure 1. However, four basic problems have been related to these pedal sensors. First, it is difficult to place and fix the sensors near the pedals in such a way that it does not disturb the driver while driving as well as to avoiding errors resulting from accidental movement of the sensors. Second, the connection of the sensors to the pedals by means of a flexible wire must be ad-hoc implemented for each car. Third, the measured displacements of the pedals are inherently very small (few centimetres) which involves incorporating a special conditioning electronic system. Finally, it’s necessary to indicate the set-up utilized to mechanically fix this type of sensors depends on the model and type of the vehicle (car, van, lorry, industrial vehicle, *etc.*). Because of the previously mentioned problems, this paper describes an approach to pedal activity estimation. The basic idea is to use the measurements of some sensors, either on-board the vehicle or added ones which are easier to deploy and adjust, to implement a feedforward artificial neural network to indirectly estimate the driver action on the control pedals.

Artificial neural networks (ANNs) are one of the most powerful tools that have been widely employed in recent years in various fields such as sensors [16–18], measurement and control [19], and engineering [20] due to their computational speed, ability to handle complex non-linear functions, robustness, and great efficiency even in cases where full information for the studied problems is absent. Function approximation is one of the basic learning tasks that an artificial neural network can accomplish. The ability of a neural network to approximate an unknown input-output mapping may be exploited in one of two ways, system identification or inverse modelling [21,22]. This paper exploits the inverse modelling capability of neural networks by estimating the pedals activity (PA) in terms of easily measurable driving variables (MV) like regime engine (RE), vehicle speed (VS), frontal inclination (FI), and linear acceleration (LA). These measurable driving signals change as a result of the driver activity on the vehicle pedals. What the authors are presenting in this paper is a proposal to deduce the pedals activity from the previously mentioned driving signals. In other words, an inverse neural network model is described in this work to estimate the vehicle pedals activity as a way to deduce driving dynamics.

**Figure 2.**
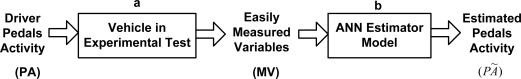
Processes involved in pedals activity estimation: (a) experimental test and (b) modelling.

The main idea of this work is to develop a mathematical model to estimate the pedals activity (*PÃ*) of any driver on the pedals of any unit of the same vehicle model using real experimental tests with particular vehicle model and several drivers. Thus, instead of directly using sensors associated with driving pedals to provide the (PA) information, the authors introduce in this paper a sensor system that includes alternative devices easier to implement and provide (MV) information of driving dynamics. Figure 2 shows these two processes for the driving pedals activity estimation followed in this work.

There are other learning machine methods that are beyond the scope of this paper to deal with function approximation problems such as support vector regression (SVR) which uses support vector machines (SVM) [23]. So this work focuses on the use of multilayer perceptron neural networks for estimating the activity of the vehicle pedals which is of special interest for traditional vehicles (cars, vans, Lorries, industrial vehicles, *etc.*) that don’t support the innovative electronic system to register the vehicle pedals signals.

The paper is arranged as follows. Section 2 shows the methodology followed to collect the PA and MV variables and visualizes the relation between them. Section 3 illustrates the inverse modelling strategy used to develop the neural models using MLP neural networks. Section 4 shows the structure of the proposed models and the results of testing them. Conclusions are included in Section 5.

## 2. Methodology

**Figure 3.**
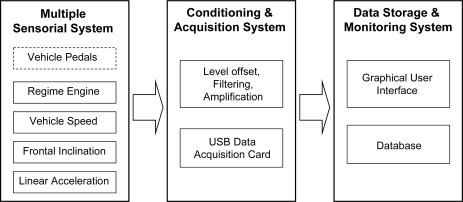
Block diagram of the measurement system.

This section describes the methodology followed to obtain the PA and MV signals. As result of previous research authors’ work [9]; an in-vehicle electronic system has been designed to measure the driver activity and its effects on driving dynamics. The designed system consists of three basic subsystems as shown in Figure 3.

From these experimental tests, the authors have selected four easily measured variables of the driving dynamics that reflect the activity on the vehicle control pedals: regime engine (RE), instantaneous velocity of the vehicle (VS), frontal inclination (FI), and linear acceleration (LA).

The first subsystem in Figure 3 includes the sensorial system responsible for measuring the required physical quantities. Among the used sensors, three potentiometers are used to provide a signal proportional to the action of the driver on the three vehicle pedals as illustrated in Figure 1 [24]. The displacement values measured by the potentiometers have been normalized between 0% and 100% of the pedal path. Moreover, an inertial measurement unit MTi from Xsens has been employed to register the frontal inclination (FI) and linear acceleration (LA) of the vehicle [25] without additional filtering [26]. The regime engine (RE) was registered by the RPM8000 sensor [27], while the instantaneous vehicle speed (VS) was measured by a GPS sensor [28]. The three sensors (*i.e.*, MTi, RPM8000, and GPS) are easy to install, don’t need special placement (except for the RPM sensor which is connected to the cigarette lighter) and their outputs are applied directly to the serial port of the PC that acts as a monitoring and storage element during the test.

The second subsystem (Figure 3) is in charge of the conditioning and acquisition of signals from sensors [29]. Finally the third subsystem is responsible for monitoring and recording the data for further offline processing. A sampling period of 20 ms, which is sufficient to represent the dynamics of the driver’s action, was chosen to capture any quick action of the driver on any of the pedals.

**Figure 4.**
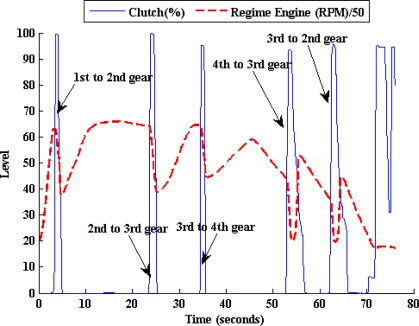
Relation between the regime engine and the clutch pedal activity.

**Figure 5.**
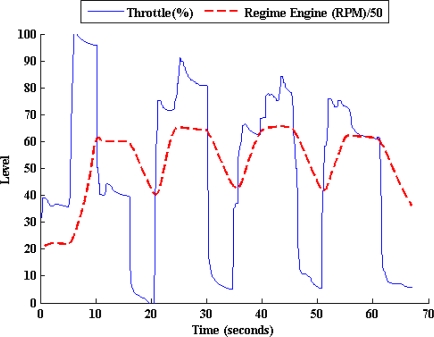
Relation between the regime engine and the throttle pedal activity (2nd gear position).

**Figure 6.**
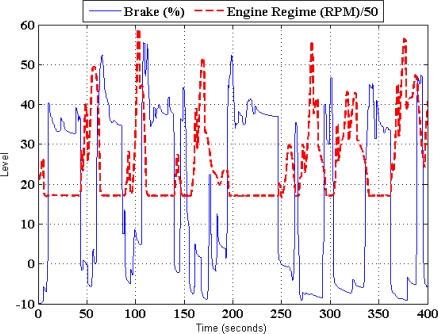
Relation between the engine regime and the brake pedal activity.

**Figure 7.**
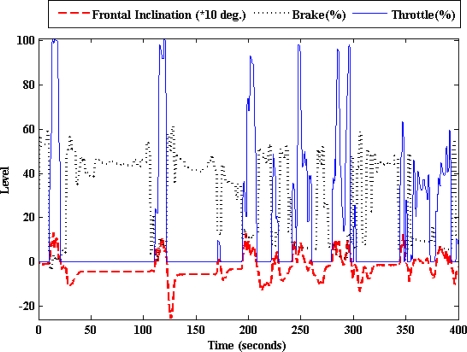
Relation between the frontal inclination and the brake and accelerator pedals activity.

**Figure 8.**
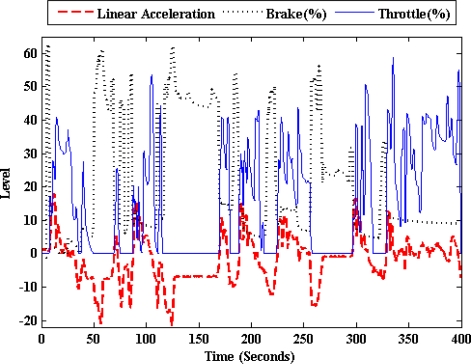
Relation between the linear acceleration and the brake and throttle pedals activity.

In Figures 4–8, experimental results obtained from a Peugeot 406 tested in both a chassis dynamometer and in an urban circuit are shown. These figures confirm the relation between the MV and the PA variables of the model to be designed. Figure 4 shows the regime engine (RE) derived from the clutch pedal. It can be appreciated that there is a clear relationship between both of these variables; one of them is activated by the driver and the other one shows the vehicle’s answer.

Figure 5 illustrates the RE deduced from the throttle pedal activity results of another laboratory experiment using the same vehicle running at second gear. As it can be seen, the regime engine increases when the throttle is pressed, however it decreases when the throttle is released (brake action is not allowed on chassis dynamometer). Figure 6 shows the results of an experiment in an urban circuit in Madrid City using the previously mentioned vehicle. It’s noted that when the foot is put on the brake pedal, the regime engine starts to decrease. As can be observed, there is a clear difference between the behaviors of the clutch, *i.e.*, on/off, and that of the throttle and brake pedals, *i.e.*, the continuous variation between two limited values of these two pedals.

Figures 7 and 8 show how the frontal inclination and linear acceleration signals of the inertial sensor vary with the throttle and brake pedals activity. In other words, the car’s chassis moves upwards and the linear acceleration increases when the throttle is pressed. On the contrary when the brake is pressed, the car’s chassis moves downwards and the linear acceleration decreases. The experimental data was obtained from the previously mentioned urban circuit using the Peugeot 406.

As a result of the previous section, the authors propose an inverse model with four input easily measurable variables (MV) and three output variables (PA). The input variables are the regime engine (RE), frontal inclination (FI), linear acceleration (LA) and vehicle speed (VS). In addition, the output variables are the estimation of the three vehicle pedals activity: throttle, brake, and clutch.

## 3. ANN Models

**Figure 9.**
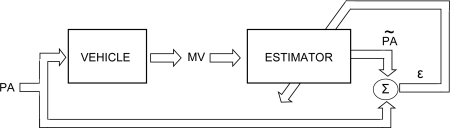
Block diagram to develop the inverse modelling system.

**Figure 10.**
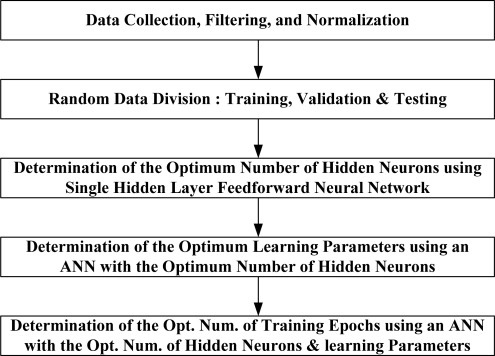
Flow chart of the ANN modeling methodology.

The inverse modelling process followed to develop the neural network estimators in this work is illustrated in Figure 9. In addition, the ANN modelling strategy applied in this work to develop the neural estimators for pedals is described in Figure 10.

Concerning the data preparation task, the early stopping technique is used to improve the generalization performance. In this technique the entire normalized data set has been divided randomly into training (80%), validation (10%), and testing (10%) subsets. The validation subset is employed during the training phase by monitoring the validation set error. It normally decreases during the initial phase of the training, as does the training set error. However when the network begins to over fit the data, the validation set error begins to rise [30]. When it is increased for a specified number of iterations (10 in this work), training is stopped and the network parameters are returned to the minimum validation set error state. The testing subset isn’t used during training but the testing set error helps compare different models based on the generalization performance.

Numerous neural networks are available for function approximation problems. A Multilayer Perceptron MLP neural network trained with backpropagation is chosen as it has many useful properties for the pedals estimation problem. It can efficiently learn large data sets as compared to Radial Basis Function RBF networks and Generalized Regression neural networks GRNN, it has been already shown to be effective for function approximation problems, it can efficiently establish a nonlinear relationship between a group of variables, it has been already used in inverse modeling processes [31], it has a relatively simple structure as compared to recurrent networks, and the backpropagation training algorithm is already implemented in well known software tools such as Matlab. The nonlinear relationship between inputs and outputs in any network requires a function that can appropriately relate them. Nonlinear transfer functions for the hidden neurons are required to introduce non-linearity into the network. It was found that an MLP network, with a sufficiently large number of neurons can satisfy the “universal approximation” property [32–35]. In this paper, MLPs with “tansig” hidden neurons and linear output neurons are investigated because it gives satisfactory results.

Since the backpropagation learning algorithm was first popularized, there have been extensive research efforts to accelerate its convergence because the basic algorithm is too slow for most practical applications. These researches fall into two categories, heuristic techniques and numerical techniques. In this work, the feedforward neural network is trained with the Levenberg-Marquardt numerical optimization method because it is the fastest for function approximation problems of networks containing up to a few hundred weights [36]. Moreover, it achieved the minimum RMSE on the testing subset as shown below in Table 1. Table 1 shows a comparison between the performances of different training algorithms. These results were obtained by training the throttle and brake MLP neural networks discussed later in this paper with 50 training epochs.

The Levenberg-Marquardt algorithm updates the network weights and biases (x vector) using the formula described in Equation (1) where J is the jacobian matrix containing first derivatives of the network errors with respect to the weights and biases, “e” is the vector of network errors, and μ is a the damping parameter:

(1) $$x_{k+1}=x_{k}-{\left[J^{T}\;J+\mu I\right]}^{-1}J^{T}\;e$$

When a particular training algorithm fails on an MLP, it could be due to one of two reasons. The learning rule fails to converge to the proper values of the network parameters, perhaps due to unsuitable network initialization. Or the inability of the given network to implement the desired function, perhaps due to a insufficient number of hidden neurons. To avoid the first possibility, the neural network models were trained and tested 10 times and the network architecture with the lowest root mean square error (RMSE) on the testing data set is chosen.

Concerning the second possibility, there is no theory yet to explain how many hidden neurons are needed to approximate any given function. If there are too few hidden neurons, a high training error and high generalization error would result due to underfitting and high statistical bias. On the other hand, if there are too many hidden neurons, there would be a low training error, but there would still be a high generalization error, due to overfitting and high variance. In most situations, there is no way to determine the best number of hidden neurons without training several networks and estimating the generalization error of each [22,30,32,34]. In this paper, the network growing technique [22] is applied by adding hidden neurons sequentially from 1 to 20 and comparing the generalization (testing) RMSE error, calculated by Equation (2), between the measured outputs and the model estimated ones. Where O is the vector of observed (measured) values, P is the vector of model-estimated values, and N is the number of samples in the testing subset. The proposed criterion in this paper is that the optimum number of hidden neurons equals that number after which the testing RMSE stops to decrease:

(2) $$\mathrm{RMSE}={\left[\frac{1}{N}\sum_{i=1}^{N}{\left(P_{i}-O_{i}\right)}^{2}\right]}^{1/2}$$

Once the optimum number of hidden neurons is obtained, a neural network with that optimum number of hidden neurons is trained several times in order to determine the best learning parameters. Finally, a neural network with the optimal number of hidden neurons and learning parameters is trained several times in order to determine the best number of training epochs. At this point, three neural network models were developed to estimate the pedals activity (*i.e.*, throttle, brake, and clutch) from the easily measured variables (regime engine, vehicle speed, linear acceleration, and frontal inclination). These models have been tested with the testing subset in order to evaluate their ability for generalization. The next section shows the testing results of these models.

Moreover a linear regression analysis was done between the model-estimated values and the real values. A model that exactly reproduces the actual observations has slope ‘a’ as 1 and intercept ‘b’ as 0. The parameters ‘a’ and ‘b’ are calculated following the least square procedure as given in Equation (3):

(3) $$\tilde{P}A=a\cdot \mathrm{PA}+b$$

## 4. Results

Below are the results of the ANN models obtained for pedals signals estimation due to the driver activity from the driving dynamics variables mentioned above. Concerning the training process of the throttle, brake, and clutch models, the maximum number of training epochs is 50 epochs; the number of patterns used in the training and testing phases are 92840 and 9284 respectively.

### 4.1. Throttle estimator

**Figure 11.**
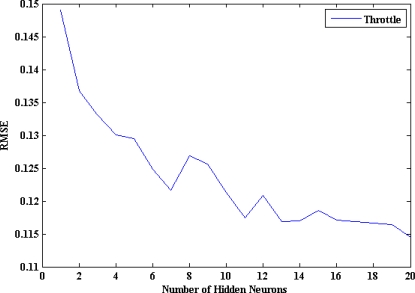
RMSE vs number of hidden neurons of the throttle pedal ANN model.

**Figure 12.**
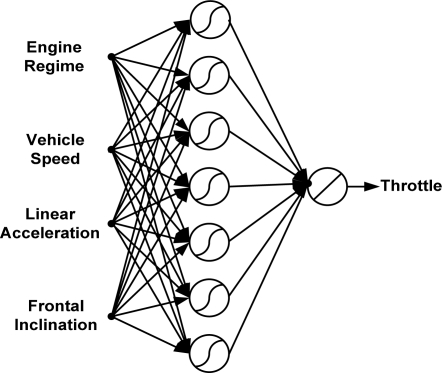
Structure of the proposed ANN model for the throttle pedal activity.

**Figure 13.**
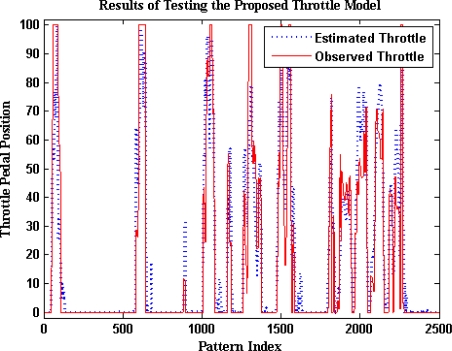
Results of testing the proposed throttle model.

The optimum number of hidden neurons is seven for the throttle pedal activity ANN model, as can be seen in Figure 11. The structure of the ANN model for the throttle pedal activity is described in Figure 12. The results of testing the optimum model of the throttle pedal activity are illustrated in Figure 13. It shows that the model estimated values follows the target values with an acceptable accuracy. The training time required to train the developed model is 75.37 s, and the testing time is 0.43 s.

**Figure 14.**
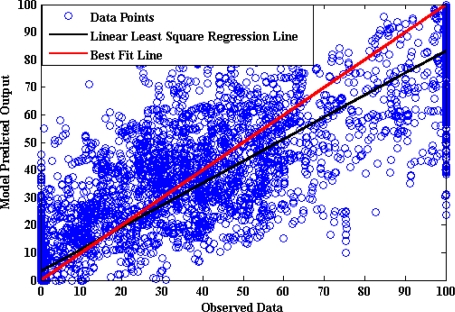
Results of linear regression analysis of the throttle model.

Moreover linear regression analysis is done between the model-estimated data and the captured data as shown in Figure 14, where the model estimations are plotted against the observations. It shows that the proposed model underestimates the throttle pedal activity when the captured values are towards the higher side and overestimate when the captured values are towards the lower side. The regression coefficient is 0.9008, a (slope) = 0.8811, b (intercept) = 3.0952.

### 4.2. Brake estimator

**Figure 15.**
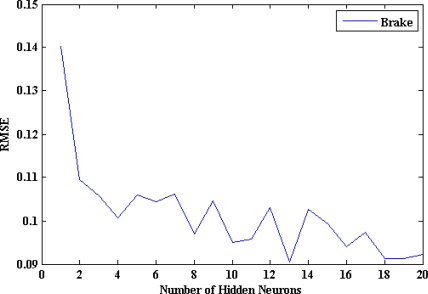
RMSE *vs.* number of hidden neurons for the brake ANN model.

**Figure 16.**
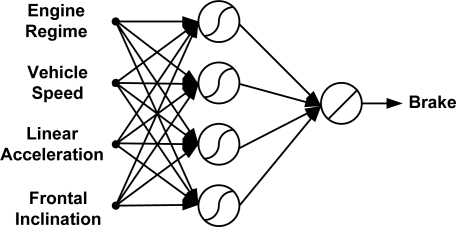
Structure of the proposed model for the brake pedal activity.

**Figure 17.**
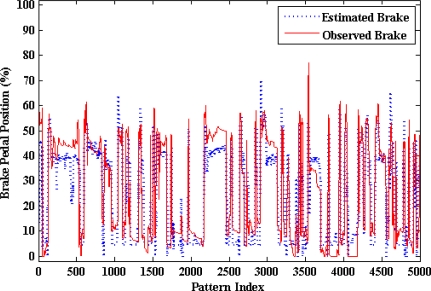
Results of testing the proposed brake model.

**Figure 18.**
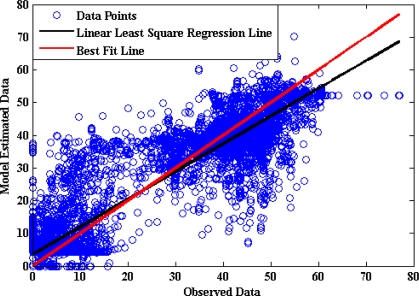
Results of linear regression analysis of the brake model.

Figure 15 shows that the optimum number of hidden neurons for the brake pedal activity ANN model is four. The internal architecture of the brake pedal model is described in Figure 16. The results from testing the proposed model for brake pedal are plotted in Figure 17 where there is a clear correspondence between the model-estimated values and the measured ones. The training time required to train the developed model is 27.44 s, and the testing time is 0.0541 s. The results of the linear regression analysis are illustrated in Figure 18. The regression coefficient is 0.9195, a (slope) = 0.846, b (intercept) = 3.6064.

### 4.3. Clutch estimator

The results of testing the ANN model of the clutch pedal developed using the same methodology as the throttle and brake, show that this prediction approach fails. Therefore the clutch pedal information has to be estimated using a different approach from that followed with the throttle and brake pedals. This is because in the case of the clutch pedal, it is not important to know the relative position of the clutch rather than the instant at which it was depressed and its continuity. This is an on/off (digital) behaviour, not a continuous behaviour like the throttle and brake, which requires different processing to obtain a good model.

**Figure 19.**
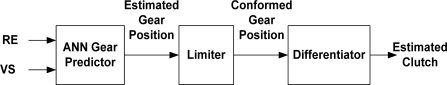
The alternative system used to model the clutch pedal activity.

An alternative approach is to use the regime engine (rpm) and vehicle speed (km/h) signals to deduce the gear position signal (discrete amplitude signal), as described in [37]. Starting from the gear position signal, the clutch activity signal can be estimated easily by detecting the instants corresponding to a gear change event. The process of predicting the gear position based on the corresponding regime engine (RE) and the vehicle speed (VS) can be achieved using ANNs. However the task of detecting the gear change events can be accomplished by a differentiator once the estimated signal is conformed. Figure 19 illustrates a block diagram of this alternative system used to model the clutch pedal activity.

**Figure 20.**
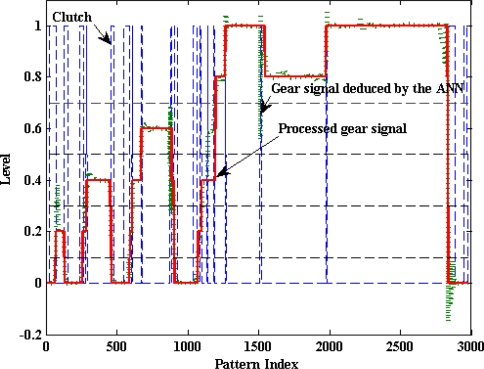
Results of testing the proposed clutch model.

Figure 20 illustrates the results of the described proposal based on data from a European Cycle Test of the Peugeot 406. The blue dashed line represents the clutch activity captured by the corresponding potentiometer. The dotted green line represents the estimated gear position signal and the solid red line represents the conformed one. As it can be appreciated, the ANN models the gear position based on the regime engine and vehicle speed with a great efficiency. Moreover, it’s straightforward that the clutch activity signal can be efficiently deduced from the gear position signal as it is shown in Figure 20.

## 5. Conclusions

The main contribution of this work is the presentation of a solution to estimate the driving activity avoiding the use of onboard sensors directly applied to the control pedals. The solution is based on an alternative sensorial system (easily measured variables), and on a neural network estimator (a specific neural model related to each pedal).

In this way, after a training period using the registered variables, the proposed system of models provides information about the driver’s activity in a simple and accurate way.

From the alternative variables point of view, observable and measurable driving signals such as the regime engine (RPM meter), vehicle speed (GPS), frontal inclination of the chassis and linear acceleration of the vehicle (inertial sensor) have been chosen. Instead of using *ad hoc* pedal sensors requiring mechanical adaptation for each vehicle, the related variables are registered by sensors that are easily and systematically installed independently of the vehicle type and model. On the other hand, they can minimally affect the driving of the vehicle contrary to the use of the conventional sensors used to capture the direct action on the vehicle pedals.

From the ANN modelling point of view, three inverse neural models based on multilayer perceptron with one hidden layer are designed. To validate the obtained models, well known criteria are utilized such as RMSE and regression analysis parameters. The model structure is valid for any vehicle (car, van, bus, lorry, industrial, *etc.*) and requires training using direct measurements of the pedals in order to determine the model parameters. The car must be tested with different drivers to determine the specific estimator for each car model.

The system has been tested with experiments using commercial vehicles, in a chassis dynamometer as well as on urban circuits; consequently this ensures the validity of the proposed models.

## Figures and Tables

**Table 1,:** Comparison between the performances of different learning algorithms.

**Training Algorithm**		**Levenberg-Marquardt**	**Scaled Conjugate Gradient**	**Resilient Backpropagation**	**BFGS Quasi-Newton**	**Variable Learning Rate**
**Testing RMSE**	**Throttle**	0.12767	0.14698	0.14245	0.13939	0.23885
	**Brake**	0.10806	0.13919	0.13629	0.13127	0.32242
